# Supplementary material for: Occupational Noise-Induced Hearing Loss among Migrant Workers in Kuwait
Source: Int J Environ Res Public Health. 2021 May 16;18(10):5295. doi: 10.3390/ijerph18105295 (PMC8156043; doi:10.3390/ijerph18105295)
Supplement: Supplementary file 1 [file ijerph-18-05295-s001.zip › ijerph-1169366-supplementary.pdf]

**Table S1.** Baseline characteristics of industrial workers according to noise exposure.

| Demographic characteristics | N | All (n = 3474) | | Not Exposed to noise (n = 2262) | | Exposed to noise (n =1212) | |
|---|---|---|---|---|---|---|---|
| | | **n** | **(%)** | **n** | **(%)** | **n** | **(%)** |
| **All** | 3474 | 3474 | (100) | 2262 | (65.1) | 1212 | (34.9) |
| Age (years) | 3474 | | | | | | |
| 21–30 | | 712 | (20.5) | 534 | (75) | 178 | (25) |
| 31–40 | | 1268 | (36.5) | 819 | (64.6) | 449 | (35.4) |
| 41–50 | | 921 | (26.5) | 564 | (61.2) | 357 | (38.8) |
| 51–60 | | 517 | (14.9) | 307 | (59.4) | 210 | (40.6) |
| ≥61 | | 56 | (1.60) | 38 | (67.9) | 18 | (32.1) |
| **Gender** | 3474 | | | | | | |
| Male | | 3434 | (98.8) | 2230 | (64.9) | 1204 | (35.1) |
| Female | | 40 | (1.2) | 32 | (80) | 8 | (20) |
| **Nationality** | 3474 | | | | | | |
| Indian | | 1974 | (56.8) | 1249 | (63.3) | 725 | (36.7) |
| Egyptian | | 483 | (13.9) | 346 | (71.6) | 137 | (28.4) |
| Bangladeshi | | 271 | (7.8) | 181 | (66.8) | 90 | (33.2) |
| Filipino | | 181 | (5.2) | 93 | (51.4) | 88 | (48.6) |
| Pakistani | | 141 | (4.1) | 86 | (61) | 55 | (39) |
| Kuwaiti | | 70 | (2.0) | 55 | (78.6) | 15 | (21.4) |
| Others | | 354 | (10.2) | 252 | (71.2) | 102 | (28.8) |
| **Experience (years)** | 3441 | | | | | | |
| ≤15 | | 2974 | (85.4) | 1947 | (65.5) | 1027 | (34.5) |
| 16–30 | | 418 | (12.1) | 262 | (62.7) | 156 | (37.3) |
| ≥30 | | 49 | (1.40) | 27 | (55.1) | 22 | (44.9) |

**Table S2.** Baseline job and industry type of industrial workers according to noise exposure status.

| Job and industry type | N | All (n = 3474) | | Not Exposed to noise (n = 2262) | | Exposed to noise (n = 1212) | |
|---|---|---|---|---|---|---|---|
| | | n | (%) | n | (%) | n | (%) |
| All | 3474 | 3474 | (100) | 2262 | (79.6) | 1212 | (20.4) |
| Job Type | 3474 | | | | | | |
| Managers | | 38 | (1.1) | 30 | (78.9) | 8 | (21.1) |
| Professionals | | 204 | (5.9) | 155 | (76) | 49 | (24) |
| Technicians and associate professionals | | 886 | (25.5) | 645 | (72.8) | 241 | (27.2) |
| Clerical support | | 110 | (3.2) | 101 | (91.8) | 9 | (8.2) |
| Services and sales | | 22 | (0.6) | 20 | (90.9) | 2 | (9.1) |
| Elementary occupations | | 735 | (21.2) | 444 | (60.5) | 291 | (39.5) |
| Crafts and related trade works | | 669 | (19.3) | 410 | (61.3) | 259 | (38.7) |
| Plant and machine operators and assemblers | | 810 | (23.3) | 457 | (56.4) | 353 | (43.6) |
| Industry type | 3474 | | | | | | |
| Mining and quarrying | | 786 | (22.6) | 484 | (61.6) | 302 | (38.4) |
| Manufacturing | | 1858 | (53.5) | 1118 | (60.2) | 740 | (39.8) |
| Water supply, sewerage, waste management, and remediation activities | | 89 | (2.6) | 89 | (100) | 0 | (0) |
| Construction | | 190 | (5.5) | 182 | (95.8) | 8 | (4.2) |
| Wholesale and retail trade and repair of vehicles and motorcycles | | 9 | (0.3) | 4 | (44.4) | 5 | (55.6) |
| Transportation and storage | | 42 | (1.2) | 27 | (64.3) | 15 | (35.7) |
| Professional, scientific, and technical activities | | 84 | (2.4) | 84 | (100) | 0 | (0) |
| Administrative and supportive service activities | | 416 | (12) | 274 | (65.9) | 142 | (34.1) |
